# Improved PSO_AdaBoost Ensemble Algorithm for Imbalanced Data

**DOI:** 10.3390/s19061476

**Published:** 2019-03-26

**Authors:** Kewen Li, Guangyue Zhou, Jiannan Zhai, Fulai Li, Mingwen Shao

**Affiliations:** 1 College of Computer and Communication Engineering, China University of Petroleum, Qingdao 266580, Shandong, China; likw@upc.edu.cn (K.L.); mwshao@upc.edu.cn (M.S.); 2 Institute for Sensing and Embedded Network Systems Engineering, Florida Atlantic University, 777 Glades Road, Boca Raton, FL 33431, USA; jzhai@fau.edu; 3 School of Geosciences, China University of Petroleum, Qingdao 266580, Shandong, China; liful@upc.edu.cn

**Keywords:** Adaptive Boosting, imbalanced data, Area Under Curve, Particle Swarm Optimization

## Abstract

The Adaptive Boosting (AdaBoost) algorithm is a widely used ensemble learning framework, and it can get good classification results on general datasets. However, it is challenging to apply the AdaBoost algorithm directly to imbalanced data since it is designed mainly for processing misclassified samples rather than samples of minority classes. To better process imbalanced data, this paper introduces the indicator Area Under Curve (AUC) which can reflect the comprehensive performance of the model, and proposes an improved AdaBoost algorithm based on AUC (AdaBoost-A) which improves the error calculation performance of the AdaBoost algorithm by comprehensively considering the effects of misclassification probability and AUC. To prevent redundant or useless weak classifiers the traditional AdaBoost algorithm generated from consuming too much system resources, this paper proposes an ensemble algorithm, PSOPD-AdaBoost-A, which can re-initialize parameters to avoid falling into local optimum, and optimize the coefficients of AdaBoost weak classifiers. Experiment results show that the proposed algorithm is effective for processing imbalanced data, especially the data with relatively high imbalances.

## 1. Introduction

Since imbalanced data can be found in any area, effective classification of imbalanced data has become critical for many applications. The classification results of imbalanced data generated by existing classification algorithms are usually significantly affected by the majority class, resulting in low accuracy in classification of the minority class. For example, the sensor network can accurately achieve target recognition under the assumption of data distribution equilibrium. However, in practical applications, the filed environment is complex and variable, and the difficulty of obtaining samples is different, which results in imbalanced data. It is easy to ignore samples of minority class in this case, resulting in incorrect classification. In the intrusion alarm application, misclassification of samples of minority class means false alarm of system, which will cause very serious consequences.

Existing approaches processing imbalanced data can be generally divided into two categories [1,2]. The first category is based on resampling at the data level, which either (i) increases the number of samples using upsampling by synthesizing new data or copying the original data, or (ii) reduces the number of samples using subsampling by extracting a small amount of data. Although resampling can improve the accuracy of minority class classification, there are some challenges. It is impossible to properly interpret the synthetic new data generated by upsampling. In addition, important information may be lost during the subsampling process. The second category is based on the ensemble and cost-sensitive approaches at the algorithm level [3,4], which increases the weights of the misclassified samples, thus improving the classification performance. The ensemble approaches that currently widely used are typically based on Boosting [5,6,7,8] or Bagging [9,10,11]. AdaBoost is a boosting algorithm and is widely used to process imbalanced data. It uses a single-layer decision tree as a weak classifier. In each training iteration, the weight of the misclassified samples generated by the previous iteration is increased, and the weight of the correctly classified samples is reduced, improving the significance of the misclassified samples in the next iteration. Although the AdaBoost algorithm can be directly used to process imbalanced data, the algorithm focuses more on the misclassified samples than samples of minority class. In addition, it may generate many redundant or useless weak classifiers, increasing the processing overhead and causing performance reduction.

Many approaches have been proposed to improve the performance of AdaBoost. Li et al. [12] proposed the BPSO-AdaBoost-KNN algorithm for multiclass imbalanced data classification, and this algorithm improves the stability of AdaBoost by effectively extracting key features. Cao et al. [13] used the gradient descent algorithm to optimize the new loss function based on the Boosting framework, and proposed the AsB and AsBL algorithms, which further verified that this approach can generate cost-sensitive classifiers with lower error cost. Yang et al. [14] used mathematical analysis and graphical methods to clarify the working principle of multiclass AdaBoost, and proposed a novel approach for processing multiclass data. This algorithm not only reduces the requirements of weak classifiers, but also ensures the effectiveness of the classification. Li et al. [15] proposed the AdaBoost composite kernel extreme learning machine, by combining the composite kernel method and the AdaBoost framework with the weighted ELM. The proposed algorithm improves performance in hyperspectral image classification. Dou et al. [16] proposed an improved AdaBoost algorithm that assigns a weight to each individual class and uses weight vectors to represent the recognition power of the base classifiers. This algorithm significantly avoids overfitting and improves classification accuracy. Xie et al. [17] proposed an ensemble evolve algorithm for imbalanced data classification by introducing the genetic algorithm to the AdaBoost algorithm. Better classifiers are generated using gene evolution and improved fitness functions, and imbalanced data classification is optimized during evolution. Guo et al. [18] treated samples of majority class that exceeded the threshold during the iteration as noise, and proposed four algorithms (i.e., A-AdaBoost, B-AdaBoost, C-AdaBoost and D-AdaBoost) based on limiting threshold growth and modifying class labels. Results show that these algorithms can effectively process imbalanced data.

In this paper, we propose AdaBoost-A, an improved AdaBoost algorithm based on AUC. The AdaBoost-A redefines the error calculation formula by introducing the AUC index into the error calculation of the weak classifier. The AUC can evaluate the performance of a classifier, and reflect the effects of imbalanced data on the classifier. As a result, the proposed AdaBoost algorithm can focus more on samples of minority class. In addition, the AdaBoost-A algorithm generates a set of weak classifiers to build a strong classifier, and the improved particle swarm optimization algorithm based on population diversity is used to further optimize the weight of the classifiers, thus decreasing the weight of redundant and useless classifiers and avoiding waste of system resources and time overhead.

The remainder of this paper is organized as follows. In Section 2, we introduce the basic principles and implementation steps of AdaBoost and Particle Swarm Optimization (PSO) algorithms. In Section 3, we illustrate the improved AdaBoost-A algorithm and ensemble algorithm PSOPD-AdaBoost-A. In Section 4, the effectiveness of PSOPD-AdaBoost-A is proved by comparison experiments with traditional AdaBoost algorithm and various improved algorithms. The conclusions are drawn in Section 5.

## 2. Background

### 2.1. Adaptive Boosting (AdaBoost)

AdaBoost (Adaptive Boosting) is an adaptive enhancement technique. It is a typical ensemble algorithm which improves classification performance by combining multiple weak classifiers into one strong classifier. In the beginning, all the samples are assigned the same weight. During the iteration, the weights of samples vary with the coefficients of weak classifiers, and the coefficients of the classifiers are calculated by the error. As a result, the AdaBoost algorithm can increase the weight of the misclassified samples and decrease the weight of the correctly classified samples. In the next iteration, the classifier will focus the misclassified samples more. Finally, all the generated weak classifiers are merged using linear combination to form a strong classifier. The steps of the AdaBoost algorithm [19] are as follows:

Input:

Training data set $T=\left\{\left(x_{1},y_{1}\right),\left(x_{2},y_{2}\right),\cdots ,\left(x_{N},y_{N}\right)\right\}$, where $x_{i}\in R^{n},y\in Y=\left\{-1,+1\right\}$, and a weak learning algorithm.

Output: 

Final classifier G(x).

1. Initialize the weight distribution of the training samples following Equation (1).
  (1) $$D_{1}=\left(w_{11},\cdots ,w_{1i},\cdots ,w_{1N}\right),\;w_{1i}=\frac{1}{N},\;i=1,2,\cdots ,N$$
  where N represents the number of samples.
2. For m=1,2,⋯,M, where M represents the number of weak classifiers.
  - Following Equation (2), get the weak classifier based on weight distribution $D_{m}.$
    (2) $$G_{m}\left(x\right)=\left\{-1,+1\right\}$$
  - Calculate the classification error rate of $G_{m}\left(x\right)$ on the training data set following Equation (3).
    (3) $$e_{m}=P\left(G_{m}\left(x\right)\neq y_{i}\right)=\overset{N}{\underset{i=1}{\sum }}w_{mi}I\left(G_{m}\left(x\right)\neq y_{i}\right)$$
  - Calculate the coefficient of $G_{m}\left(x\right)$ following Equation (4).
    (4) $$\alpha_{m}=\frac{1}{2}log\frac{1-e_{m}}{e_{m}}$$
  - Update the weight distribution of the training samples following Equations (5)–(7).
    (5) $$D_{m+1}=\left(w_{m+1,1},\cdots ,w_{m+1,i},\cdots ,w_{m+1,N}\right)$$
    (6) $$w_{m+1,i}=\frac{w_{mi}}{Z_{m}}\exp\left(-\alpha_{m}y_{i}G_{m}\left(x_{i}\right)\right)$$
    where $Z_{m}$ is the normalization factor.
    (7) $$Z_{m}=\overset{N}{\underset{i=1}{\sum }}w_{mi}\exp\left(-\alpha_{m}y_{i}G_{m}\left(x_{i}\right)\right)$$
3. Build a linear combination of basic classifiers and get the final classifier G(x) following Equations (8) and (9).
  (8) $$f\left(x\right)=\overset{M}{\underset{m=1}{\sum }}\alpha_{m}G_{m}\left(x\right)$$
  (9) $$G\left(x\right)=\mathrm{sign}\left(f\left(x\right)\right)=\mathrm{sign}\left(\overset{M}{\underset{m=1}{\sum }}\alpha_{m}G_{m}\left(x\right)\right)$$

The advantages of the AdaBoost algorithm are summarized as follows. (1) The AdaBoost algorithm can use various weak classifiers without filtering features. In addition, it delivers high execution efficiency, and can avoid overfitting issues. (2) The AdaBoost algorithm trains the weak classifiers without knowing the prior knowledge. The synthetic strong classifier can significantly improve the classification accuracy, and it is suitable for classification of most types of data. (3) The training of rough weak classifiers is much easier than training of the accurate strong classifiers. It trains multiple weak classifiers to form a strong classifier with better classification performance.

### 2.2. PSO

PSO was proposed by James Kenney and Russ Eberhart in 1995 [20]. The algorithm is derived from the study of predation behavior of birds, and it is a method based on iteration. Imagine a scene where there is a piece of food in a certain area and a group of randomly distributed birds are searching for the food. They obtain their distances from the food, but do not get the specific location of the food. The best way to solve this problem is to change the flight path based on the current location of the bird closest to the food and flight experience of each bird, to locate the food.

The PSO algorithm considers each solution as a bird, called a particle. Each particle has an adaptive value that represents the current state of its own solution. In each iteration, each particle adjusts its moving direction and velocity based on the global optimal solution and the optimal solution found by the particle itself, and gradually approaches the optimal particle.

The basic principle of the standard particle swarm algorithm is as follows [21].

Suppose that there are m particles searching for the optimal solution in an N-dimensional target space and randomly initialize the position and velocity of each particle following Equations (10)–(12). Where the vector $U_{i}$ represents the position of particle i, and the vector $V_{i}$ represents the flight speed of particle i.

(10) $$U_{i}=\left(u_{i1},u_{i2},\ldots ,u_{iN}\right)$$

(11) $$V_{i}=\left(v_{i1},v_{i2},\ldots ,v_{iN}\right)$$

(12) i=1,2,…,m

As Equation (13) shows, the current best position $P_{i}$ found by particle i is:

(13) $$P_{i}=\left(p_{i1},p_{i2},\ldots ,p_{iN}\right)$$

As Equation (14) shows, the current best location $P_{gbest}$ found by all particles is:

(14) $$P_{gbest}=\left(p_{g1},p_{g2},\ldots ,p_{gN}\right)$$

The position and velocity of particle i is then updated following Equations (15) and (16).

(15) $$v_{in}^{k+1}=\omega v_{in}^{k}+c_{1}\cdot rand()\cdot \left(p_{in}-u_{in}^{k}\right)+c_{2}\cdot rand()\cdot \left(p_{gn}-u_{in}^{k}\right)$$

(16) $$u_{in}^{k+1}=u_{in}^{k}+v_{in}^{k+1}$$

where ω is the inertia weight, $c_{1},c_{2}$, two positive constant, are the acceleration factors, $v_{in}^{k+1}$ represents the nth-dimensional velocity component generated by the (k+1)th iteration of the ith particle, and $u_{in}^{k+1}$ represents the nth-dimensional position component generated by the (k+1)th iteration of the ith particle. The position and velocity update formula is divided into three parts. The first part is the inertia part, which indicates the particle’s degree of trust in its own speed. The second part is the self-cognitive part, which indicates the particle’s degree of trust in its own experience. The third part is the social cognitive part, which indicates the degree of trust in the best adaptive particle [22].

Characteristics of PSO algorithm can be summarized as [23]:

1. It is possible to quickly approximate the optimal solution and achieve effective optimization of parameters.
2. It is suitable for searching within the scope of continuity and solving the maximum and minimum problems of continuous functions.
3. It is easy to implement with low complexity and requires a small number of parameters.
4. It is easy to fall into local optimum.

## 3. The Proposed Approach

### 3.1. Area Under Curve (AUC)

Confusion matrix is the common method to reflect performance of classification model. Taking a two-class model as an example, the confusion matrix of this model is calculated as shown in Table 1.

Based on the confusion matrix, the Accuracy, Precision, Recall and F1-Measure are defined as follows:

(17) $$\mathrm{Accuracy}=\frac{TP+TN}{TP+FP+TN+FN}$$

(18) $$\mathrm{Precision}=\frac{TP}{TP+FP}$$

(19) $$\mathrm{Recall}=\frac{TP}{TP+FN}$$

(20) $$F1=\frac{2\times Precision\times Recall}{Precision+Recall}=\frac{2TP}{2TP+FP+FN}$$

where TP is the number of true positives, which represents cases that the positive class are correctly classified. Where FN is the number of false negatives, which represents cases that the positive class are classified as negative. Where TN is the number of true negatives, which represents cases that the negative class are correctly classified. Where FP is the number of false positives, which represents cases that negative class are classified as positive.

The TP, FP, TN, and FN measures can be collected to construct a plot, which is a Receiver Operating Characteristic (ROC) curve, which the true positive rate (TPR) as the ordinate and the false positive rate (FPR) as the abscissa. The calculation formula TPR and FPR are shown in Equation (21).

(21) $$\mathrm{TPR}=\frac{TP}{TP+FN},\;\mathrm{FPR}=\frac{FP}{TN+FP}$$

The value of AUC is the area under the ROC curve. Suppose 1−s and r are the probabilities of FP and TP, respectively. The AUC is estimated by Equation (22), where $\Delta \left(1-s\right)={\left(1-s\right)}_{\gamma }-{\left(1-s\right)}_{\gamma -1}$, $\Delta r=r_{\gamma }-r_{\gamma -1}$ and γ is an index.

(22) $$\mathrm{AUC}=\underset{\gamma }{\sum }\left\{\left[r_{\gamma }\cdot \Delta \left(1-s\right)\right]+\frac{1}{2}\left[\Delta r\cdot \Delta \left(1-s\right)\right]\right\}$$

AUC is a comprehensive evaluation of classification models, which can provide more useful information than accuracy measurement.

### 3.2. The AdaBoost-A Algorithm

Although the AdaBoost algorithm can be directly applied to imbalanced data, the ensemble algorithm pays more attention to the misclassified samples, rather than samples of minority class. According to the error calculation formula of the weak classifier of AdaBoost, the error is only related to the weight and the number of misclassified samples. There is no additional processing for the misclassified samples of minority class, so the AdaBoost ensemble algorithm is not well suited for processing imbalanced data [24]. To solve this challenge, we propose an improved AdaBoost algorithm (AdaBoost-A) that introduces the AUC [25] into the error function calculation. At the algorithm level, the error rate metric cannot properly reflect the performance of the classifier. For example, there are 90 samples in class A and 10 samples in class B. If classifier divides all test samples into class A, the error rate of classifier is 10%. However, it is clear that this classifier makes no sense. As the area under the ROC curve, AUC can effectively reflect the comprehensive performance of the classifier. If the classifier is biased towards majority class classification, the AUC of the classifier will be very small, and 1-AUC will be very large. The error is determined by combining the product of classification error rate and 1-AUC, which can effectively improve the classification accuracy of AdaBoost. The improved error calculation is shown in Equation (23).

(23) $$e_{m}=2\left(1-\mathrm{AUC}\right)\cdot P\left(G_{m}\left(x\right)\neq y_{i}\right)=2\left(1-\mathrm{AUC}\right)\cdot \overset{N}{\underset{i=1}{\sum }}w_{mi}I\left(G_{m}\left(x\right)\neq y_{i}\right)$$

where $e_{m}$ represents error rate of the mth weak classifer, $G_{m}\left(x\right)$ is the mth weak classifer, $y_{i}$ represents the actual label of the ith sample, $w_{mi}$ represents the weight corresponding to the ith sample in the mth iteration.

### 3.3. The PSOPD-AdaBoost-A Ensemble Algorithm

Although the AdaBoost algorithm can combine multiple weak classifiers into one strong classifier, the coefficients of the weak classifiers are determined in the iteration process. These coefficients cannot be changed later, so it is inevitable to generate redundant or useless weak classifiers that have large weights. This can significantly affect the readability of the classifiers and increase system overhead. To overcome these shortcomings, our approach uses the PSO algorithm to optimize the weights of the weak classifiers of AdaBoost-A. This algorithm assigns large weights to the weak classifiers with high accuracy, and small weights to the redundant or useless weak classifiers, further improving the accuracy and readability of AdaBoost classifier. 

PSO is an optimization algorithm with a small number of parameters and fast convergence, but it is easy to fall into local optimum [26]. Therefore, this paper proposes an ensemble algorithm by improved PSO based on population diversity optimizing AdaBoost-A (PSOPD-AdaBoost-A). It can further optimize the coefficient weights of the weak classifiers of AdaBoost-A by performing re-initialization when it falls into in local optimum. The proposed improvements focus on using the error function of AdaBoost-A as the fitness function, and adopting the standard PSO algorithm to optimize the weights of the weak classifiers of AdaBoost-A. If the optimal particle does not change for ten consecutive iterations, the optimal particle is retained, and the position and velocity of other particles are reinitialized. The iteration is continued until the configured number of iterations is reached. The optimal particle does not change in multiple iterations, and it is likely to fall into local optimum. By re-initialization, the search range of the particle is enlarged, and the population diversity is enhanced. At the same time, the optimal particle is retained during re-initialization to avoid loss of the optimal solution of the population.

The PSOPD-AdaBoost-A ensemble algorithm is described as follows:

1. Use the AdaBoost-A algorithm to generate several (M) weak classifiers, and the coefficients of the weak classifiers are expressed following Equation (24).
  (24) $$A=\left(a_{1},a_{2},\ldots ,a_{M}\right)\;k=1,2,\ldots ,M$$
  where $a_{k}$ represents the weight coefficient of the kth weak classifier.
2. Set the population size to m and randomly initialize the position and velocity of each particle following Equations (25)–(27).
  (25) $$U_{i}=\left(u_{i1},u_{i2},\ldots ,u_{iM}\right)$$
  (26) $$V_{i}=\left(v_{i1},v_{i2},\ldots ,v_{iM}\right)$$
  (27) i=1,2,…,m
3. Use the position component of each particle as the weight coefficient of the weak classifier of AdaBoost-A. As Equation (28) shows, the error rate $e_{i}$ of AdaBoost-A is calculated as the fitness value of each particle.
  (28) $$e_{i}=2\left(1-\mathrm{AUC}\right)*\overset{Q}{\underset{s=1}{\sum }}I\left(\mathrm{sign}\left(\overset{M}{\underset{k=1}{\sum }}u_{ik}G_{k}\left(x\right)\right)\neq y_{s}\right)$$
  where Q represents the number of samples, $e_{i}$ represents the error rate of the ith particle, and $y_{s}$ represents the true class label of the sth sample.
4. For each particle, the fitness value generated by each iteration is compared with the fitness value of the optimal position passed by the particle. If the fitness value is greater than the fitness value of the optimal position, the current position is taken as the optimal location passed by the particle, recorded as $P_{i}$.
5. For each particle, the fitness value generated by each iteration is compared with the fitness value of the optimal position passed by all particles. If the fitness value is greater than the fitness value of the optimal position of all particles, the current position is taken as the global optimal location, recorded as $P_{gbest}$.
6. Update the position and velocity of the particle in the following iteration based on the Equations (15) and (16).
7. When the maximum number of iterations is reached or the error is small enough, the iteration stops. Otherwise, check the number of consecutive times that the optimal particle remains unchanged. If it reaches the threshold (10 is used in our configuration), the optimal particle is retained, and the position and velocity of other particles are reinitialized. If it is less the threshold, no action is performed. Then continue to execute steps 4–6.

## 4. Evaluation

### 4.1. Test Data

We evaluate the proposed algorithm using the Vehicle, Horse Colic, Ionosphere and Statlog imbalanced datasets from UCI repository and KC1, JM1, PC3, PC5, CM1 imbalanced datasets from NASA. In addition, the weak classifiers are generated by Decision-Stump. Table 2 lists the details of the nine imbalanced datasets used in the evaluation. The label bad in Ionosphere is considered to be a minority class, and the label good in Ionosphere is considered to be a majority class. The label 1 in Statlog is considered to be a minority class, and other labels in Statlog are considered as a majority class. The label van in Vehicle is considered to be a minority class, and labels saab, bus, and opel in Vehicle are considered as a majority class.

### 4.2. Analysis of the AdaBoost-A Algorithm

**Figure 1:**
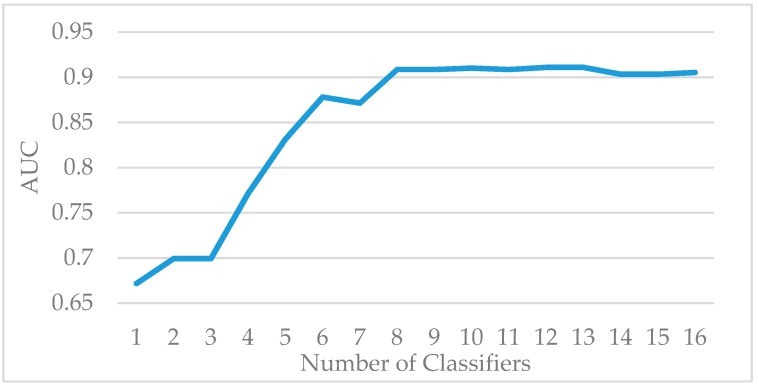
The AUC of AdaBoost Algorithm on Vehicle Training Set.

**Figure 2:**
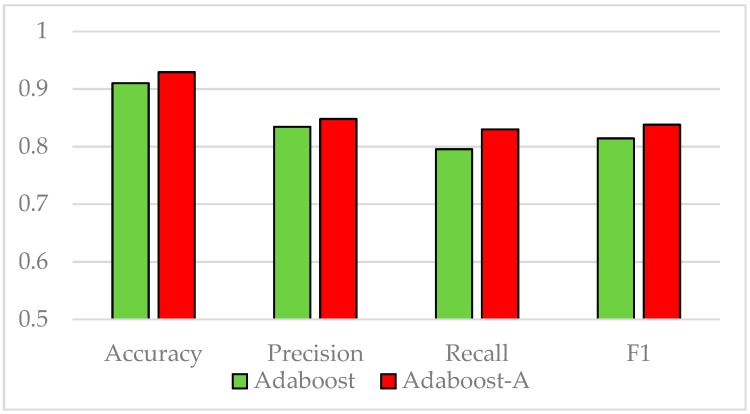
Performance Comparison on Vehicle Test Set.

The Vehicle dataset is split into training and test sets at a ratio of 7:3. The standard AdaBoost algorithm is used to classify the samples in the training set. As the number of weak classifiers increases, the growth trend of AUC is shown in Figure 1. When the number of weak classifiers reaches 10, the increase of the evaluation index AUC significantly slows down, indicating that increasing the number of weak classifiers hardly improves the AUC. Therefore, the number of weak classifiers in the experiments is set to 10. Figure 2 shows the comparison of accuracy, precision, recall, and F1 value of the standard AdaBoost algorithm and the AdaBoost-A algorithm on the Vehicle test set. Results show that the AdaBoost-A algorithm achieves 92.9% accuracy, 84.8% precision, 83% recall, and 83.8% F1 value, and the standard AdaBoost algorithm achieves 91.0% accuracy, 83.4% precision, 79.5% recall, and 81.4% F1 value. The proposed algorithm not only improves the overall accuracy, but also reduces the error of minority class classification.

**Figure 3:**
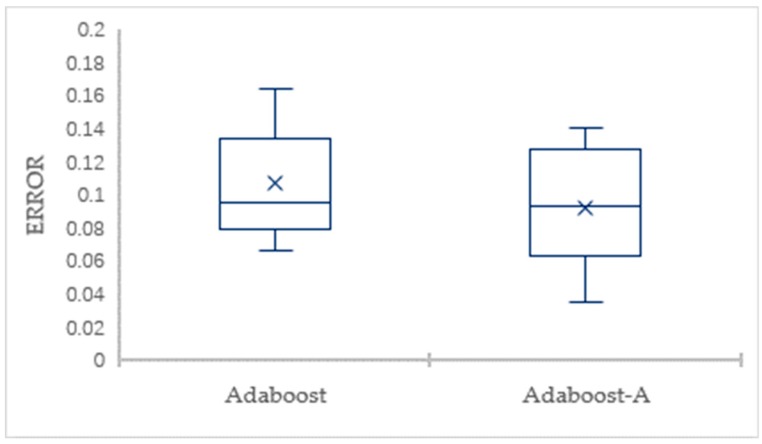
The Error Comparison of AdaBoost and AdaBoost-A on Vehicle Dataset.

**Figure 4:**
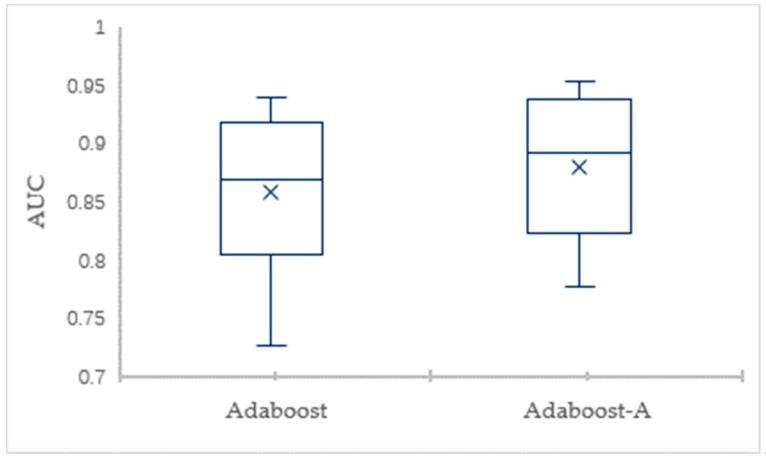
The AUC Comparison of AdaBoost and AdaBoost-A on Vehicle Dataset.

To eliminate the impact of data division and guarantee valid results, the 10-fold CV is employed to evaluate the classification performance. The detailed comparison results for the AdaBoost-A algorithm and the AdaBoost algorithm on Vehicle dataset in terms of the error and AUC are showed through box plots in Figure 3 and Figure 4, respectively. Figure 3 shows that the maximum, minimum, and average of AdaBoost-A algorithm is lower than the AdaBoost algorithm in terms of error. Figure 4 shows that the maximum, minimum, and average of AdaBoost-A algorithm is higher than the AdaBoost algorithm in terms of AUC.

**Figure 5:**
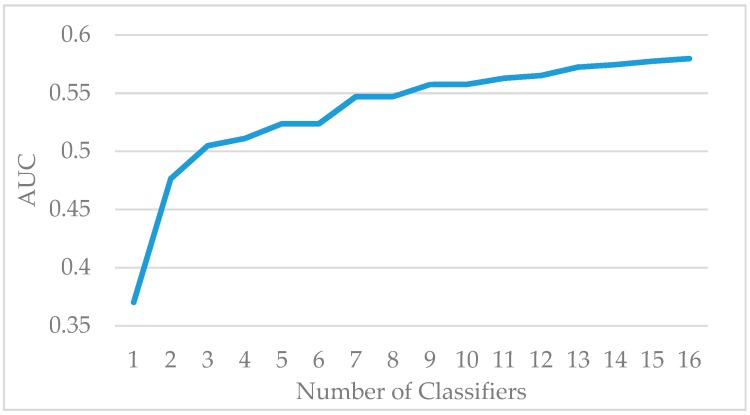
The AUC of AdaBoost Algorithm on KC1 Training Set.

**Figure 6:**
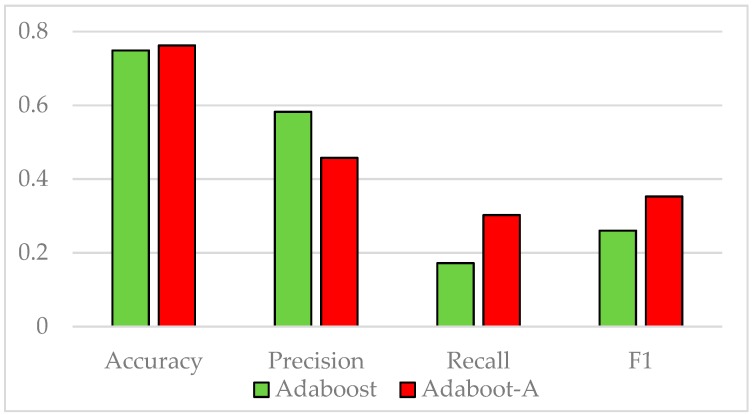
Performance Comparison on KC1 Test Set.

The KC1 dataset is split into training and test sets at a ratio of 7:3. The standard AdaBoost algorithm is used to classify the samples in the training set. As the number of weak classifiers increases, the growth trend of AUC is shown in Figure 5. When the number of weak classifiers reaches 10, the increase of the evaluation index AUC significantly slows down. Therefore, the number of weak classifiers in this experiment is set to 10. Figure 6 shows the comparison of accuracy, precision, recall, and F1 value of the standard AdaBoost algorithm and the AdaBoost-A algorithm on the KC1 test set. Results show that the AdaBoost-A algorithm achieves 76.2% accuracy, 45.8% precision, 30.2% recall, and 35.3% F1 value, and the standard AdaBoost algorithm achieves 74.9% accuracy, 58.2%precision, 17.2% recall, and 26% F1 value.

**Figure 7:**
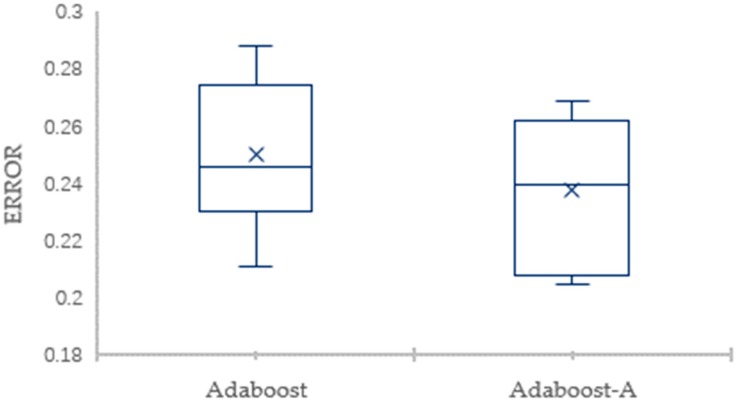
Error Comparison of AdaBoost and AdaBoost-A on KC1 Dataset.

**Figure 8:**
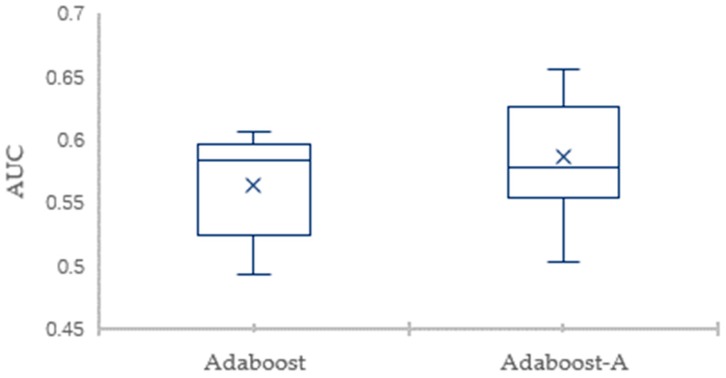
The AUC Comparison of AdaBoost and AdaBoost-A on KC1 Dataset.

The detailed comparison results of the 10-fold CV for the AdaBoost-A algorithm and the AdaBoost algorithm on KC1 dataset in terms of the error and AUC are showed through box plots in Figure 7 and Figure 8, respectively. Figure 7 shows that the maximum, minimum, and average of AdaBoost-A algorithm is lower than the AdaBoost algorithm in terms of error. Figure 8 shows that the maximum, minimum, and average of AdaBoost-A algorithm is higher than the AdaBoost algorithm in terms of AUC.

Through the above experiments, it is proved that the proposed AdaBoost-A algorithm is more effective than AdaBoost algorithm.

### 4.3. Analysis of the PSOPD-AdaBoost-A Ensemble Algorithm

**Figure 9:**
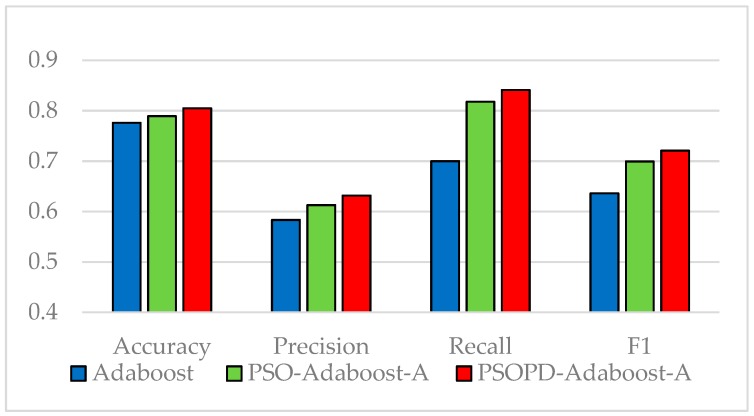
Performance Comparison of the AdaBoost, PSO-AdaBoost-A, and PSOPD-AdaBoost-A on Horse Colic Dataset.

**Figure 10:**
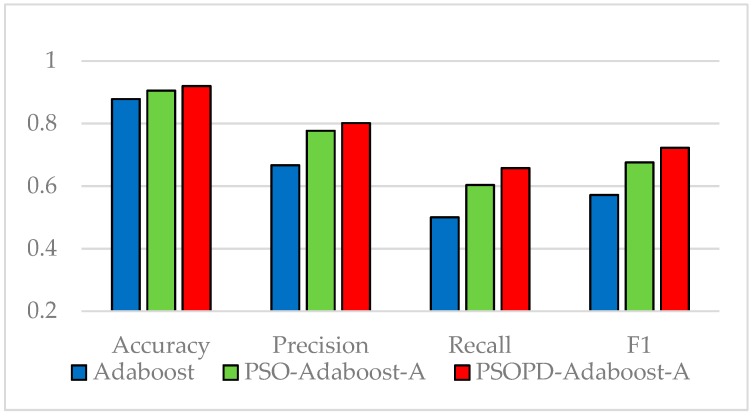
Performance Comparison of the AdaBoost, PSO-AdaBoost-A, and PSOPD-AdaBoost-A on Ionosphere Dataset.

**Figure 11:**
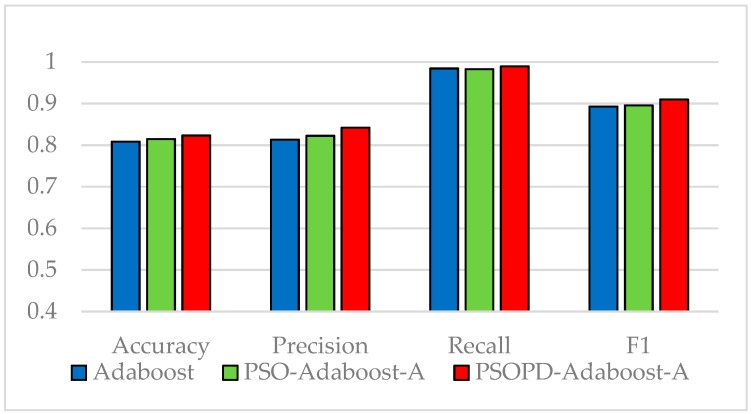
Performance Comparison of the AdaBoost, PSO-AdaBoost-A, and PSOPD-AdaBoost-A on JM1 Dataset.

**Figure 12:**
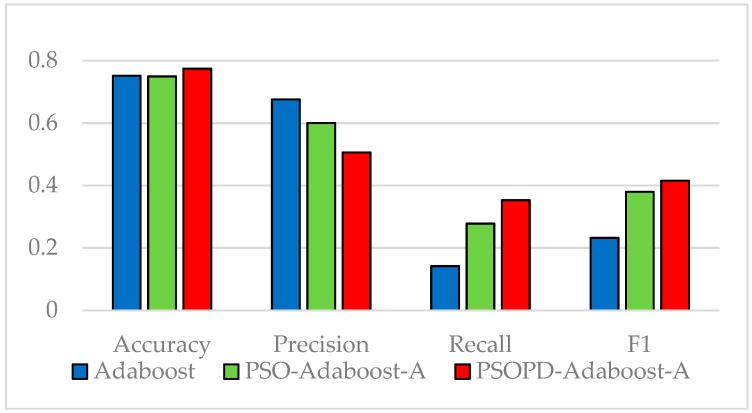
Performance Comparison of the AdaBoost, PSO-AdaBoost-A, and PSOPD-AdaBoost-A on KC1 Dataset.

**Figure 13:**
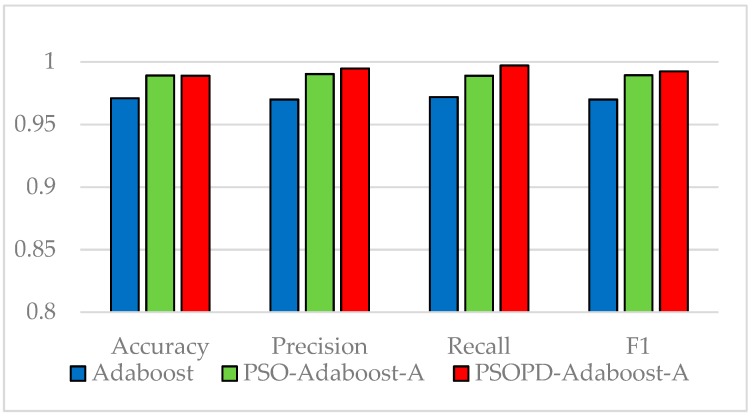
Performance Comparison of the AdaBoost, PSO-AdaBoost-A, and PSOPD-AdaBoost-A on Statlog Dataset.

The coefficients of AdaBoost-A weak classifiers are optimized by the improved PSO based on population diversity and the standard PSO on the five imbalanced datasets, respectively. We compare classification performance of them by performing 5-fold CV. The detailed classification results of the AdaBoost, PSO-AdaBoost-A, and PSOPD-AdaBoost-A algorithms based on the average of 100 runs are showed in Figure 9, Figure 10, Figure 11, Figure 12 and Figure 13.

As shown in Figure 9, Figure 10, Figure 11, Figure 12 and Figure 13, the classification performance of the PSO-AdaBoost-A and PSOPD-AdaBoost-A ensemble algorithms is much higher than the AdaBoost algorithm. It illustrates that optimizing the weight coefficients of AdaBoost weak classifiers can significantly improve the performance of the classifiers. The PSOPD-AdaBoost-A algorithm achieves 80.4% accuracy, 63.2% precision, 84.1% recall, and 72.1% F1 value on the Horse Colic dataset, which is higher than that of the PSO-AdaBoost-A classifier. The PSOPD-AdaBoost-A algorithm achieves 92.0% accuracy, 80.2% precision, 65.8% recall, and 72.2% F1 value on the Ionosphere dataset, which is higher than that of the PSO-AdaBoost-A classifier. The PSOPD-AdaBoost-A algorithm achieves 82.3% accuracy, 84.2% precision, 99.0% recall, and 91.0% F1 value on the JM1 dataset, which is higher than that of the PSO-AdaBoost-A classifier. The PSOPD-AdaBoost-A algorithm achieves 77.5% accuracy, 50.6% precision, 35.3% recall, and 41.6% F1 value on the KC1 dataset, which is higher than that of the PSO-AdaBoost-A classifier in terms of accuracy, recall, and F1 value. The PSOPD-AdaBoost-A algorithm achieves 98.9% accuracy, 99.5% precision, 99.7% recall, and 99.3% F1 value on the Statlog dataset, which is higher than that of the PSO-AdaBoost-A classifier in terms of precision, recall, and F1 value. The experimental results presented above show that the improved PSO algorithm based on population diversity can effectively avoid falling into local optimum and achieve higher classification accuracy, and prove that the PSOPD-AdaBoost-A algorithm is effective in processing imbalanced data.

### 4.4. Comparison the PSOPD-AdaBoost-A and Other Improved Algorithms

To solve the imbalance problem, researchers have proposed many approaches to improve the ensemble algorithms, but most of the improved methods are still sensitive to the relatively high imbalance rate. Next, we compare classification performance of our PSOPD-AdaBoost-A approach and boosting algorithms including G-AdaBoost based on genetic algorithm [17], B-AdaBoost based on label modification and D-AdaBoost based on weight limitation [18], bagging algorithms including Random Forest and Extra Trees, sampling method including Smote-based AdaBoost by performing 5-fold CV on the Vehicle, PC3, PC5, and CM1 datasets. For a fair comparison, the number of weak classifiers of algorithms for experiment mentioned above is set to 10, and the weak classifier is generated by Decision-Stump. Results show that the PSOPD-AdaBoost-A ensemble algorithm is effective on datasets with relatively high imbalance rates.

**Figure 14:**
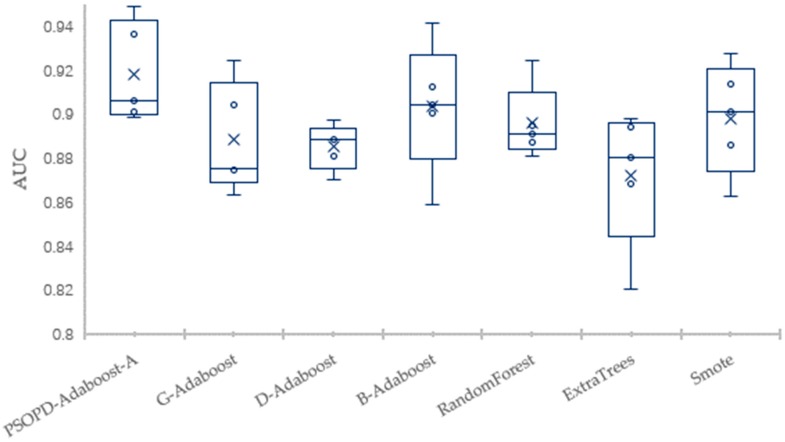
Comparison of AUC on the Vehicle Dataset.

**Figure 15:**
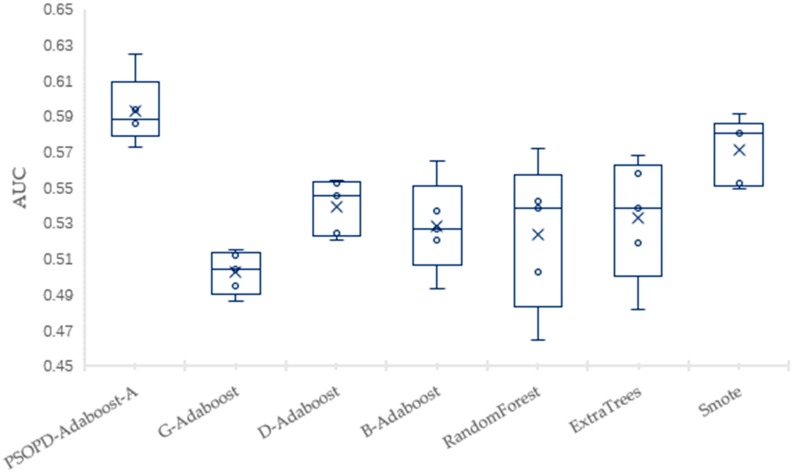
Comparison of AUC on the PC3 Dataset.

**Figure 16:**
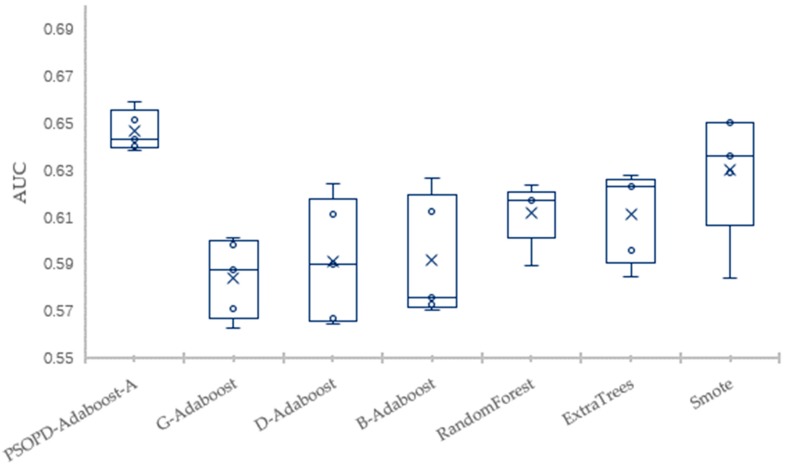
Comparison of AUC on the PC5 Dataset.

**Figure 17:**
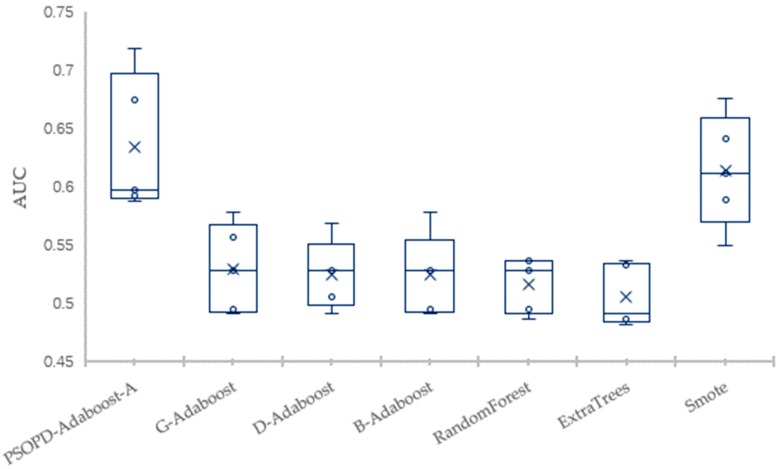
Comparison of AUC on the CM1 Dataset.

The mean of Accuracy, Precision, Recall, F1, AUC, and Error of the four datasets are summarized in Table 3, Table 4, Table 5 and Table 6, respectively. The largest values are highlighted in bold for each performance measure in each table. To further verify the effectiveness of PSOPD-AdaBoost-A ensemble algorithm for processing imbalanced data, the AUC values of each run are showed through box plots in Figure 14, Figure 15, Figure 16 and Figure 17.

Table 3 shows that the PSOPD-AdaBoost-A method achieves the highest performance of the seven comparison algorithms in terms of accuracy, F1 value, and AUC classifying the Vehicle dataset, its precision is slightly lower than the G-AdaBoost algorithm, and its recall is slightly lower than the Smote method. Figure 14 shows that the maximum, minimum, and average of PSOPD-AdaBoost-A algorithm is the highest among seven algorithms in terms of AUC, demonstrating the effectiveness of the PSOPD-AdaBoost-A algorithm in classifying the Vehicle dataset.

Table 4 shows that the PSOPD-AdaBoost-A method achieves the highest performance of the seven comparison algorithms in terms of accuracy, precision, F1 value and AUC classifying the PC3 dataset, and its recall is lower than the Smote method. Figure 15 shows that the maximum, minimum, and average of PSOPD-AdaBoost-A algorithm is the highest among seven algorithms in terms of AUC, demonstrating the effectiveness of PSOPD-AdaBoost-A in classifying the PC3 dataset.

Table 5 shows that the PSOPD-AdaBoost-A method achieves the highest performance of the seven comparison algorithms in terms of precision, F1 value, and AUC classifying the PC5 dataset, its accuracy is slightly lower than the Extra Trees algorithm, and its recall is slightly lower than the Smote method. Figure 16 shows that the maximum, minimum, and average of PSOPD-AdaBoost-A algorithm is the highest among seven algorithms in terms of AUC, demonstrating the effectiveness of PSOPD-AdaBoost-A in classifying the PC5 dataset.

Table 6 shows that the PSOPD-AdaBoost-A method achieves the highest performance of the seven comparison algorithms in terms of accuracy, precision, F1 value and AUC classifying the CM1 dataset, and its recall is lower than the Smote method. Figure 17 shows that the maximum, minimum, and average of PSOPD-AdaBoost-A algorithm is the highest among seven algorithms in terms of AUC, demonstrating the effectiveness of PSOPD-AdaBoost-A in classifying the CM1 dataset.

Through the above comparative experiments, it is proved that the PSOPD-AdaBoost-A ensemble algorithm is more effective in processing imbalanced data compared to many improved algorithms.

## 5. Conclusions

Traditional AdaBoost algorithm focuses on the misclassified samples instead of the samples of minority class. In this paper, we propose an improved AdaBoost algorithm (AdaBoost-A). Since the AUC can effectively reflect the performance of the classifier, we introduce the AUC into error calculation, making the AdaBoost focus more on the classification accuracy of the minority. Furthermore, the AdaBoost algorithm may generate redundant or useless weak classifiers, significantly affecting the readability of the classifier. We propose an ensemble algorithm, PSOPD-AdaBoost-A, which can further optimize the weight of the weak classifiers. Experimental results show that the AdaBoost-A and PSOPD-AdaBoost-A ensemble algorithms can effectively classifying imbalanced datasets, Vehicle, KC1, Horse Colic, Ionosphere, JM1, and Statlog. Next, we compare the imbalanced data classification performance of PSOPD-AdaBoost-A with ensemble algorithms including G-AdaBoost, B-AdaBoost, D-AdaBoost, Random Forest, and Extra Trees, sampling method including Smote, and four datasets with relatively high imbalance rate, Vehicle, PC3, PC5, and CM1 are used in the comparison. The results show that the PSOPD-AdaBoost-A ensemble algorithm is effective in processing data with relatively high imbalance rate compared to other improved algorithms. Our future work is dedicated to applying the proposed algorithm to the field of sensors, accurately achieving classification of targets by processing imbalanced data acquired by sensors.

## Figures and Tables

**Table 1:** Confusion matrix.

		Predicted Class	
		Positive	Negative
Actual class	Positive	TP	FN
	Negative	FP	TN

**Table 2:** Details of the Five Imbalanced Datasets.

Dataset	The Number of Samples	Majority Class	Minority Class	Imbalance Ratio (IR)
Vehicle	846	647	199	3.25:1
KC1	1497	1183	314	3.76:1
Horse Colic	368	227	141	1.61:1
Ionosphere	351	225	126	1.79:1
JM1	10,878	8776	2102	4.17:1
Statlog	2310	1980	330	6:1
PC3	1077	943	134	7.04:1
PC5	1711	1240	471	2.63:1
CM1	505	457	45	10.2:1

**Table 3:** Comparison of Classification Results on the Vehicle Dataset.

Algorithm	Accuracy	Precision	Recall	F1	AUC	Error
PSOPD-AdaBoost-A	0.925000	0.809345	0.902400	0.851406	0.917187	0.074999
G-AdaBoost	0.923584	0.861940	0.811999	0.833173	0.885012	0.076415
D-AdaBoost	0.924529	0.857178	0.824000	0.836553	0.889777	0.075471
B-AdaBoost	0.914150	0.781936	0.892000	0.831131	0.906493	0.085849
Random Forest	0.911886	0.841605	0.806001	0.823128	0.872567	0.088114
Extra Trees	0.920377	0.831528	0.84800	0.838903	0.896098	0.079633
Smote	0.897169	0.708473	0.960000	0.814594	0.898271	0.102831

**Table 4:** Comparison of Classification Results on the PC3 Dataset.

Algorithm	Accuracy	Precision	Recall	F1	AUC	Error
PSOPD-AdaBoost-A	0.859704	0.414426	0.248235	0.310944	0.593736	0.140296
G-AdaBoost	0.856293	0.142857	0.047058	0.091314	0.509970	0.143707
D-AdaBoost	0.859293	0.357936	0.111764	0.165239	0.539780	0.140707
B-AdaBoost	0.854075	0.267125	0.094115	0.135947	0.528838	0.145925
Random Forest	0.854074	0.207045	0.113529	0.136262	0.524223	0.145936
Extra Trees	0.854322	0.242409	0.125294	0.164234	0.532223	0.145677
Smote	0.737777	0.208130	0.506405	0.294673	0.572923	0.262223

**Table 5:** Comparison of Classification Results on the PC5 Dataset.

Algorithm	Accuracy	Precision	Recall	F1	AUC	Error
PSOPD-AdaBoost-A	0.737662	0.581946	0.455764	0.511665	0.647268	0.262336
G-AdaBoost	0.744060	0.575478	0.238983	0.339432	0.591601	0.255940
D-AdaBoost	0.744392	0.577383	0.249152	0.3460050	0.591027	0.255607
B-AdaBoost	0.739719	0.560215	0.257627	0.3486072	0.590426	0.260280
Random Forest	0.747196	0.545823	0.403875	0.463364	0.612219	0.252904
Extra Trees	0.749532	0.552212	0.403389	0.466045	0.613078	0.250468
Smote	0.650000	0.414715	0.624235	0.498326	0.631312	0.350000

**Table 6:** Comparison of Classification Results on the CM1 Dataset.

Algorithm	Accuracy	Precision	Recall	F1	AUC	Error
PSOPD-AdaBoost-A	0.896553	0.344151	0.355000	0.349376	0.634760	0.103464
G-AdaBoost	0.865620	0.281204	0.204555	0.236418	0.526376	0.138880
D-AdaBoost	0.867637	0.340035	0.10666	0.161439	0.525507	0.123463
B-AdaBoost	0.850210	0.250256	0.126086	0.167212	0.526376	0.140788
Random Forest	0.894060	0.262445	0.190000	0.220776	0.517173	0.103938
Extra Trees	0.885784	0.343360	0.173333	0.229055	0.506231	0.110236
Smote	0.752755	0.226427	0.666666	0.335852	0.614239	0.247245
